# The carbon‐quality temperature hypothesis: Fact or artefact?

**DOI:** 10.1111/gcb.16539

**Published:** 2022-11-30

**Authors:** Lìyǐn L. Liáng, Miko U. F. Kirschbaum, Vickery L. Arcus, Louis A. Schipper

**Affiliations:** ^1^ Manaaki Whenua − Landcare Research Palmerston North New Zealand; ^2^ Te Aka Mātuatua ‐ School of Science University of Waikato Hamilton New Zealand

**Keywords:** carbon‐quality temperature hypothesis, enzyme‐catalysed reactions, global warming, microbial decomposition, soil organic carbon, temperature dependence

## Abstract

Climate warming can reduce global soil carbon stocks by enhancing microbial decomposition. However, the magnitude of this loss remains uncertain because the temperature sensitivity of the decomposition of the major fraction of soil carbon, namely resistant carbon, is not fully known. It is now believed that the resistance of soil carbon mostly depends on microbial accessibility of soil carbon with physical protection being the primary control of the decomposition of protected carbon, which is insensitive to temperature changes. However, it is still unclear whether the temperature sensitivity of the decomposition of unprotected carbon, for example, carbon that is not protected by the soil mineral matrix, may depend on the chemical recalcitrance of carbon compounds. In particular, the carbon‐quality temperature (CQT) hypothesis asserts that recalcitrant low‐quality carbon is more temperature‐sensitive to decomposition than labile high‐quality carbon. If the hypothesis is correct, climate warming could amplify the loss of unprotected, but chemically recalcitrant, carbon and the resultant CO_2_ release from soils to the atmosphere. Previous research has supported this hypothesis based on reported negative relationships between temperature sensitivity and carbon quality, defined as the decomposition rate at a reference temperature. Here we show that negative relationships can arise simply from the arbitrary choice of reference temperature, inherently invalidating those tests. To avoid this artefact, we defined the carbon quality of different compounds as their uncatalysed reaction rates in the absence of enzymes. Taking the uncatalysed rate as the carbon quality index, we found that the CQT hypothesis is not supported for enzyme‐catalysed reactions, which showed no relationship between carbon quality and temperature sensitivity. The lack of correlation in enzyme‐catalysed reactions implies similar temperature sensitivity for microbial decomposition of soil carbon, regardless of its quality, thereby allaying concerns of acceleration of warming‐induced decomposition of recalcitrant carbon.

## Graphical abstract

It has long been hypothesised that resistant low‐quality carbon is more temperature‐sensitive to decomposition than labile high‐quality carbon (the carbon‐quality temperature hypothesis). Here, we identify an unavoidable artefact inherent in previous tests of the hypothesis when carbon quality is defined as the decomposition rate at an arbitrarily chosen reference temperature. Instead, we defined the carbon quality of different compounds as their uncatalysed reaction rates. Our results showed no correlation between carbon quality and temperature sensitivity of enzyme‐catalysed reactions, therefore, did not support the hypothesis. It suggests similar temperature sensitivity of biological decomposition of soil organic matter regardless of its quality.

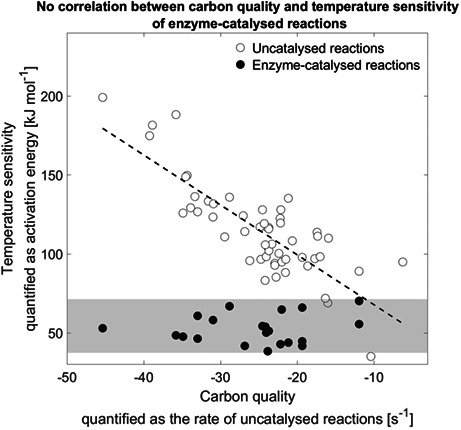

## 1 INTRODUCTION

Soils store twice as much carbon in the upper 1 m as the atmosphere (Batjes, 1996). These large carbon stocks in soil organic matter (SOM) could be lost through enhanced decomposition under a warming climate, leading to a positive soil carbon‐climate feedback (García‐Palacios et al., 2021; Kirschbaum, 2000). The extent of the enhancement of SOM decomposition in response to increasing temperature depends on the temperature sensitivity of decomposition. However, our understanding of temperature sensitivity (Sierra, 2012), for example, the temperature coefficient *Q*_10_, of SOM decomposition, is still incomplete (Davidson et al., 2000; Fang et al., 2005; Giardina & Ryan, 2000; Knorr et al., 2005; Melillo et al., 2002). This key uncertainty limits our ability to predict how SOM decomposition may respond to climate change.

Much of our understanding of the temperature response of SOM decomposition is based on studies of short‐term measurements of soil respiration rates that are dominated by the response of readily decomposable labile carbon. The bulk of the carbon stocks in the soil, however, consists of resistant carbon that decomposes more slowly, and these rates may respond to temperature differently from those for labile carbon (Bosatta & Ågren, 1999; Davidson et al., 2000; Davidson & Janssens, 2006; Hartley et al., 2021; Knorr et al., 2005). It is, therefore, uncertain whether insights from the responses of the turn‐over of labile carbon can be applied to the ultimately more important turn‐over rates of resistant soil carbon. This uncertainty hinders our ability to predict the overall impacts of climate warming on SOM decomposition rates (Conant et al., 2011).

An important obstacle to understanding the temperature sensitivity of SOM decomposition lies in the confounding of responses between the two principal stabilisation mechanisms of organic carbon in the soil, that is, chemical recalcitrance and physical protection by the matrix of soil minerals (Dungait et al., 2012). To avoid semantic confusion, hereafter the word “resistance” or “resistant” refers to the slow decomposition of SOM irrespective of its causes, that is, either controlled by physical protection or its chemical properties. And the word “recalcitrance” or “recalcitrant” only refers to the chemical properties of SOM. Traditionally, the molecular structure of the SOM molecules, or chemical recalcitrance, had been thought to be the primary factor to determine the decomposition rates in soils (Melillo et al., 1982; Sollins et al., 1996; von Lützow & Kögel‐Knabner, 2009). For example, lignin in litter or the soil is regarded as a recalcitrant compound due to its complex chemical structure and thermodynamically stable molecular configuration. Its breakdown is, therefore, expected to require a higher activation energy than that of simple molecules like glucose (Davidson & Janssens, 2006).

However, newer work has indicated that the resistance to degradation of SOM is mostly controlled by the interaction between soil minerals and organic carbon molecules (Conant et al., 2011; Dungait et al., 2012; Vogel et al., 2015) because of the formation of organo‐mineral complexes that can protect organic carbon from microbial decomposition (Baldock & Skjemstad, 2000; Eusterhues et al., 2005; Kleber et al., 2015). The physical protection is, therefore, now generally considered to be more important for protecting soil carbon than chemical recalcitrance (Dungait et al., 2012; Kirschbaum et al., 2020; Marschner et al., 2008; Mikutta et al., 2006), or, as Schmidt et al. (2011) described it that “the persistence of soil organic carbon is primarily not a molecular property, but an ecosystem property”. The formation of organo‐mineral complexes in soils can render chemically labile molecules resistant to decomposition. The resistance of SOM decomposition, therefore, involves not only the chemical recalcitrance of carbon compounds but also their accessibility (Conant et al., 2011; Dungait et al., 2012) to microbes and their extracellular enzymes (Allison et al., 2018). The physical protection of organic carbon, or their encapsulation in particle aggregates in soils, is also likely to respond less to temperature than unprotected carbon (Hartley et al., 2021; Moinet et al., 2018).

Nonetheless, ongoing research continues to investigate whether there are any general patterns between the biochemical recalcitrance of organic matter and its temperature sensitivity (Alves et al., 2021; Briones et al., 2021; Li et al., 2017; Liu et al., 2022; Moinet & Millard, 2020; Park et al., 2022; Reynolds et al., 2017). The role of biochemical recalcitrance attains particular importance under conditions where physical protection is necessarily less important, such as in organic soils, in the litter layer or in soils with limited mineral protective capacity like sandy soils.

**FIGURE 1:**
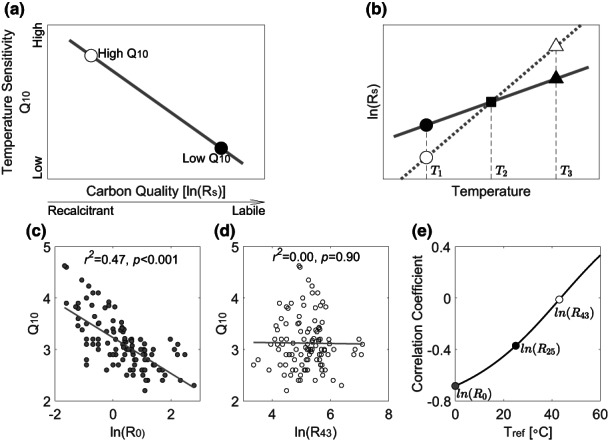
Correlations between *Q*_10_ and the logarithm of respiration rate, ln(*R*_s_). (a) Diagram of the carbon‐quality temperature hypothesis, showing a recalcitrant carbon compound with a higher *Q*_10_ compared with that for a labile carbon compound, resulting in a negative relationship between *Q*_10_ and carbon quality. (b) Temperature response curves of respiration rate for two hypothetical carbon compounds. Reanalyses of a global data set of soil incubation experiments, shown in (c), give a significant negative correlation between *Q*_10_ and ln(*R*_0_), the respiration rate at 0°C, but (d), no correlation between *Q*_10_ and ln(*R*_43_), the respiration rate at 43°C, and (e), the changes of correlation coefficient between *Q*_10_ and ln(*R*_s_) at chosen reference temperatures from 0 to 60°C. The soil respiration data set came from Fierer et al. (2006) and Li et al. (2017).

Biochemically recalcitrant SOM decomposes more slowly because of its complex and thermodynamically stable molecular structure that may also make its decomposition more sensitive to temperature (as indicated by a higher *Q*_10_) than that of labile compounds (Figure 1a). This effect on the decomposition of recalcitrant compounds can be expressed through a higher activation energy than that of labile compounds (Davidson & Janssens, 2006), which would suggest that chemically recalcitrance compounds should respond more strongly to temperature than more labile compounds. This notion has been theoretically formalised as the carbon‐quality temperature (CQT) hypothesis (Bosatta & Ågren, 1999). If the CQT hypothesis is correct, global warming would then lead to larger carbon losses from SOM decomposition and enhanced positive climate feedback through enhanced decomposition of any large stores of biochemically recalcitrant carbon.

Experimental tests of the CQT hypothesis have generally tried to assess whether there is an inverse relationship between a temperature sensitivity index, *Q*_10_ (Fierer et al., 2005, 2006; Li et al., 2017; Mikan et al., 2002; Xu et al., 2012) or activation energy, *E*_a_ (Craine et al., 2010) and a carbon quality index (Figure 1a). Determining carbon quality, however, is problematic as studies have not usually been able to chemically characterise diverse mixtures of substrates in the soil or incorporate the effects of physical protection (Dungait et al., 2012) within the soil matrix.

Carbon quality, therefore, has often been defined functionally as the respiration rate at a common reference temperature. Different studies have used either exponential or Arrhenius‐like functions to derive temperature‐sensitivity and carbon quality indices by using measurements of soil respiration rate (*R*_s_) at different temperatures (*T*). For example, in the exponential function, *R*_s_ = *R*_0_e^*bT*^ or its log‐transformed version ln(*R*_s_) = ln(*R*_0_) *+ bT*, the respiration rate at 0°C, that is, ln(*R*_0_), is often defined as the carbon quality index (e.g., Fierer et al., 2005, 2006; Li et al., 2017; Mikan et al., 2002; Xu et al., 2012), and the parameter *b* is used to determine the temperature sensitivity index *Q*_10_ (*Q*_10_ = e^10*b*^). Using this approach with data from soil incubation experiments, different researchers (Fierer et al., 2005, 2006; Li et al., 2017; Mikan et al., 2002; Xu et al., 2012) have reported negative correlations between *Q*_10_ and ln(*R*_0_) to support the CQT hypothesis (Figure 1a).

The validity of the negative correlation between *Q*_10_ and ln(*R*_0_) has, however, been challenged on statistical grounds, since a correlation between *Q*_10_ and ln(*R*_0_) could simply arise from random measurement errors (Reichstein et al., 2005). As *b* is the slope and ln(*R*_0_) is the intercept of the regression of ln(*R*_s_) against temperature, any random variation in individual data points would have inverse effects on fitted ln(*R*_0_) and *b* values (Reichstein et al., 2005). To counter that, others have argued that this error‐originated compensation between slope and intercept could be overcome if many independent samples were used (Fierer et al., 2006) where the randomness of slopes and intercepts might mitigate against any consistent pattern between them. In particular, data from different geographical locations were collected to justify CQT by demonstrating a common negative correlation between *Q*_10_ (Fierer et al., 2006) or activation energy (Craine et al., 2010) and the carbon quality index that was defined as the respiration rate at a reference temperature. Despite concern about the analysis raised previously (Reichstein et al., 2005), ongoing studies continue to frequently apply reasoning based on the CQT hypothesis to interpret temperature responses of soil carbon decomposition (Ghosh et al., 2020; Li et al., 2017; Liu et al., 2022; Yang et al., 2022; Yanni et al., 2020). It is thus warranted to reappraise the validity of experimental tests of the CQT hypothesis.

## 2 A CONCEPTUAL PARADOX OF CURRENT EXPERIMENTAL TESTS

In addition to the statistical problem discussed by Reichstein et al. (2005), we found that the definition of carbon quality index, that is, the respiration rate at an arbitrarily chosen reference temperature, even results in a conceptual paradox, as we demonstrate below. If recalcitrant carbon has a lower decomposition rate at a reference temperature, as postulated by the CQT hypothesis (Figure 1b), it must inevitably mean that both labile and recalcitrant carbon must have the same decomposition rates at a cross‐over temperature because of the difference in slopes for the corresponding ln(*R*_s_)–*T* curves (Figure 1b).

For example, at *T*_1_, ln(*R*_s_) of a labile carbon compound (Figure 1b, closed circle) is higher than that of a recalcitrant carbon compound (Figure 1b, open circle). Since the recalcitrant compound has a higher *Q*_10_, and thus a steeper slope of its ln(*R*_s_)–*T* curve (Figure 1b, dashed line), the two curves must cross at *T*_2_ (squares, the cross‐over temperature). At an even higher temperature *T*_3_, the carbon compound that was defined as more recalcitrant at *T*_1_, would be defined as more labile at *T*_3_. The combination of the CQT, together with an arbitrary choice of reference temperature, thus results in the paradoxical conclusion that a given compound can be defined as either more labile or more recalcitrant just by changing the reference temperature. This means that simply changing the reference temperature from low to high to define carbon quality could shift the correlation between *Q*_10_ and ln(*R*_s_) from negative to positive.

## 3 REANALYSIS OF A GLOBAL DATA SET OF SOIL INCUBATION EXPERIMENTS

Figure 1b presents a purely hypothetical case. To determine whether this paradox is also evident in a realistic set of observations, we reanalysed a data set of temperature response measurements of soil respiration that had been compiled from 113 independent soil incubations across 60 locations globally, consisting of 77 incubation experiments in the United States (Fierer et al., 2006) and 36 incubations in China (Li et al., 2017). Experimental setups and sampling methods have been described in detail in the original papers. Briefly, soils were sampled from various ecosystem types at different latitudes globally and were incubated in the laboratory to determine the respiration rate between 10 and 30°C (Fierer et al., 2006) or between 4 and 28°C (Li et al., 2017). By assuming a constant *Q*_10_ over the measured temperatures, an exponential function was applied to describe the temperature dependence of soil respiration as:

$$R_{s}=R_{0}e^{\mathrm{bT}}\Rightarrow \ln\left(R_{s}\right)=\ln\left(R_{0}\right)+\mathrm{bT}$$

where *R*_s_ is respiration rate, *R*_0_ and *b* are fitted parameters. In the semilogarithmic plot of ln(*R*_s_) versus *T* (°C), ln(*R*_0_) is the logarithm of respiration rate at 0°C and *b* is the slope of the linear regression of ln(*R*_*s*_) versus *T*. The parameter *b* further defines *Q*_10_, a temperature sensitivity index of soil respiration as *Q*_10_ = e^10*b*^. Using the reported *Q*_10_ and ln(*R*_0_), we reconstructed the temperature response curve for each individual incubation and calculated respiration rates at temperatures ranging from 0 to 60°C, which is within the relevant range for biological reactions. Using the recalculated respiration rate, we further determined correlations between *Q*_10_ and carbon quality, defined as the logarithm of respiration rate at temperatures chosen between 0 and 60°C at 1°C increments. Data were processed and plotted using MATLAB R2018a (The MathWorks, Inc.).

If carbon quality is defined as the logarithm of soil respiration rate at 0°C, this set of observations results in a significant negative correlation between *Q*_10_ and ln(*R*_0_) (Figure 1c). This would be consistent with the CQT hypothesis. However, the choice of 0°C as the reference temperature is arbitrary, and if one, instead, chose the respiration rate at 43°C, ln(*R*_43_), as the proxy for quality index, the correlation between the quality index and *Q*_10_ would disappear (Figure 1d), and the result would then be inconsistent with the CQT hypothesis. More generally, the correlation between *Q*_10_ and ln(*R*) can shift from negative to positive simply by arbitrarily choosing different reference temperatures from 0 to 60°C (Figure 1e).

This dependence of the correlation coefficient on a selected reference temperature clearly presents a conceptual problem for testing the CQT hypothesis within temperatures ranging from 0 to 60°C for soil respiration. The respiration rate would only represent a valid carbon quality index if the negative relationship between *Q*_10_ and the carbon quality index remained irrespective of the chosen reference temperature, within the relevant temperature range. However, this requirement is not met (Figure 1b–e), since the observation of a negative correlation, as the central tenet of the CQT hypothesis, depends entirely on the arbitrary choice of a reference temperature for determining the carbon quality index. Tests of the CQT hypothesis, therefore, require alternative measures of carbon quality.

## 4 THE CHEMICAL DEFINITION OF CARBON QUALITY

Carbon quality intrinsically refers to the degree of difficulty of a carbon compound being decomposed through a chemical reaction. This degree of difficulty of a reaction may be defined as the spontaneous reaction rate in an aqueous solution in the absence of catalysts such as enzymes (Wolfenden, 2006), namely the uncatalysed rate, *k*_non_. A small *k*_non_ value means a slow reaction rate and thus a difficult reaction and a recalcitrant compound. For example, the hydrolysis of phosphate monoester dianions, like fructose‐1,6‐bisphosphate (a critical compound in carbohydrate catabolism in biological systems), is one of the slowest uncatalysed biological reactions (Lad et al., 2003). This reaction has a *k*_non_ value of 2.0 × 10^−20^ s^−1^ at 25°C, corresponding to a half‐life of 1.1 × 10^12^ years (Lad et al., 2003). Because of the generally slow nature of uncatalysed reactions, it is a common practice to estimate *k*_non_ at ambient temperatures (Wolfenden, 2006) by measuring reaction rates at a series of greatly elevated temperatures and then extrapolating reaction rates back to ambient temperature, for example, 25°C, using a linear Arrhenius plot (Figure S1).

## 5 TESTING THE CQT HYPOTHESIS IN UNCATALYSED AND ENZYME‐CATALYSED REACTIONS

To test whether a negative relationship, as the central tenet of the CQT hypothesis, exists between *Q*_10_ or *E*_a_ and *k*_non_ as the carbon quality index, we collected data from a total of 56 uncatalysed and 21 corresponding enzyme‐catalysed reactions from the published literature. The activation energy (*E*_a_) of reactions was determined by the slope (*−E*_a_/*R*) of the temperature response curve in the Arrhenius plot (see an example in Figure S1). In data sets where the slope of the temperature response curve was given as enthalpy of activation (−*ΔH* ^‡^/*R*) by fitting the Eyring equation instead of the Arrhenius function, we determined *E*_a_ as *E*_a_ = *ΔH* ^‡^ *+ RT* (Chang & Thoman, 2014), where *R* is the universal gas constant. For uncatalysed reactions, both *E*_a_ and the reaction rate at 25°C (*k*_25_) were directly collected from tables published in the literature. For enzyme‐catalysed reactions, *E*_a_ was collected from tables, text or recalculated from graphs of published studies.

To determine the correlation coefficients between *Q*_10_ and *k*_non_ at different temperatures, *k*_non_ at temperatures from 0 to 200°C was calculated by using *E*_a_ and the respective rates at 25°C. We further applied the Jackknife resampling technique (Martinez & Martinez, 2015) to calculate the correlation coefficients for both uncatalysed (*n* = 56) and the corresponding enzyme‐catalysed reactions (*n* = 21) by repeatedly omitting one observation from the original data set. Therefore, there were 56 and 21 estimates of the correlation coefficients at each temperature for uncatalysed and catalysed reactions, respectively. The mean correlation coefficient and the associated standard errors at each temperature were further determined based on the calculated correlation coefficients from Jackknife resampling (Martinez & Martinez, 2015).

**FIGURE 2:**
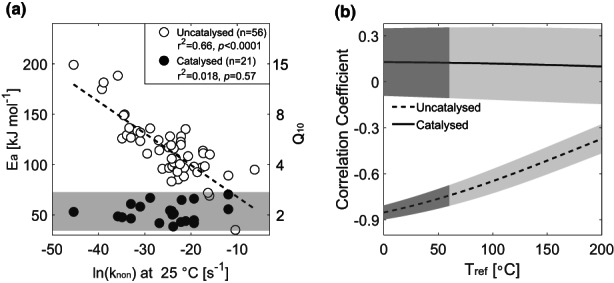
Correlation between activation energy (*E*_a_) and the logarithm of uncatalysed rates, ln(*k*_non_), used here as the carbon quality index. (a) The correlation between ln(*k*_non_) at 25°C and *E*_a_ of uncatalysed (open circles) and the corresponding enzyme‐catalysed (closed circles) reactions. The right *y*‐axis shows the corresponding *Q*_10_ values calculated between 20 and 30°C. The shaded band indicates the range of *E*_a_ or *Q*_10_ for enzyme‐catalysed reactions. (b) The correlation coefficient between *E*_a_ and ln(*k*_non_) at different reference temperature from 0 to 200°C. The light‐shaded areas show the standard errors of the estimated correlation coefficients for both reactions using the jackknife resampling technique. The dark‐shaded areas indicate the biological temperature range of 0–60°C.

From this data collection of uncatalysed reactions, we found that the *k*_non_ of different reactions at 25°C spanned a range from 10^−3^ to 10^−20^ s^−1^ corresponding to half‐lives from minutes to billions of years. Correspondingly, the activation energies of uncatalysed reactions ranged from 35 to 199 kJ mol^−1^, with *Q*_10_, calculated between 20 and 30°C, ranging from 1.6 to 15. In this analysis of uncatalysed reactions, we also found a significant negative correlation between *Q*_10_ and ln(*k*_non_) at 25°C (Figure 2a), similar to the correlation between ln(*R*_*0*_) and *Q*_10_ described for soil respiration (Figure 1c). However, in contrast to the inconsistent correlation shown in Figure 1e for soil respiration, we obtained a consistent negative correlation between observed rates and *E*_a_ or *Q*_10_ over the relevant biological temperature range, for example, 0 to 60°C, and also over a much wider temperature range of up to 200°C (Figure 2b). Even this correlation must eventually be lost at much higher temperatures (Figure S2) according to the conceptual paradox shown in Figure 1b. For both uncatalysed reactions and soil respiration, it is impossible to avoid the conceptual paradox. If one defines carbon quality as a rate at an arbitrarily chosen temperature, it must change the quality assessment with the choice of reference temperature. A derived negative correlation, therefore, cannot be used to support the CQT hypothesis for either soil respiration or uncatalysed reactions.

However, it is possible to test the CQT hypothesis for enzyme‐catalysed reactions by using the corresponding uncatalysed rate, i.e., ln(*k*_non_), as the carbon quality index and *Q*_10_ derived from enzyme‐catalysed reactions. Since ln(*k*_non_) and *Q*_10_ of catalysed reactions are two independent variables derived from separate measurements, it avoids any problems of circularity in the derived correlation, thus providing a valid test of the CQT hypothesis for catalysed reactions. For enzyme‐catalysed reactions, we found that there was no correlation between *E*_a_ and ln(*k*_non_) at 25°C (Figure 2a; Figure S3), or at any chosen reference temperature between 0 and 200°C (Figure 2b). This lack of correlation between *Q*_10_ of enzyme‐catalysed reactions and ln(*k*_non_), therefore, does not support the CQT hypothesis for enzyme‐catalysed reactions like microbial decomposition of SOM. Instead, our results suggest that under conditions where decomposition rates are controlled by enzymatic process, the temperature sensitivity should be similar regardless of the difference in chemical recalcitrance of the degradable compounds.

## 6 THE CATALYTIC POWER OF ENZYMES

For enzyme‐catalysed reactions, all *E*_a_ values remained within the relatively narrow range from about 40 to 70 kJ mol^−1^, with no apparent consistent correlation with the quality of the reactants involved. Compared to a much wider range of values from about 90 to 200 kJ mol^−1^in uncatalysed reactions, this narrow range of *E*_a_ for enzyme‐catalysed reactions is also supported by a comprehensive synthesis on the universality of enzymatic rate–temperature dependencies that showed a consistent *Q*_10_ across hundreds of enzymes (Elias et al., 2014).

**FIGURE 3:**
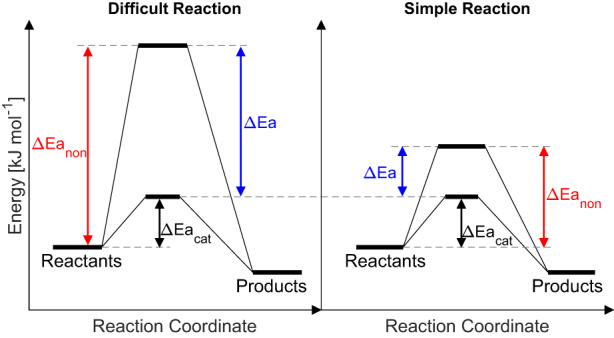
Energy diagram for difficult and simple reactions. In the absence of enzymes, a difficult reaction with recalcitrant substrates needs to overcome a higher energy barrier (∆Ea_non_) than that of simple reactions. Enzymes have evolved structures that can lower the energy barriers (∆Ea_cat_) of both difficult and simple reactions to similar levels through different rate enhancements (*k*_cat_/*k*_non_ or *∆*Ea).

Indeed, enzymes typically catalyse reactions at time scales of seconds at biological temperatures, even for reactions that have half‐times of millions of years without support by catalysts (Radzicka & Wolfenden, 1995). Enzymes can achieve this by lowering the energy barrier or *E*_a_ of the uncatalysed reactions (Figure 3). The rates of enzyme‐catalysed reactions (*k*_cat_) generally range only about 10^4^‐fold (seconds to hours, Wolfenden, 2011) while the rates of their corresponding uncatalysed reactions (*k*_non_) can vary 10^19^‐fold from 10^−1^ to 10^−20^ s^−1^. This implies vastly different rate enhancements (*k*_cat_/*k*_non_) in catalysing chemical reactions by different enzymes (Radzicka & Wolfenden, 1995). For enzymes to be able to function meaningfully and productively within current Earth's environment, they must be able to lower the energy barriers of their reactions to similar levels (Figure 3; Figure S3; Table S1), irrespective of the original energy barriers of uncatalysed reactions. For the catalysis of different reactions, enzymes, therefore, must have been able to achieve much greater efficiency enhancements and lowering *E*_a_ by much greater amounts than for simpler reactions (Figure 3; Figure S3). This would have also coincidentally resulted in the loss of *E*_a_ vs. ln(*k*_non_) or *Q*_10_ vs. ln(*k*_non_) correlations that we observed in the uncatalysed reactions (Figure 2).

We need to re‐emphasise that for biological processes, like SOM decomposition that involve enzyme‐catalysed reactions, the catalytic power of enzymes can overcome the physico‐chemical constraints of uncatalysed reactions as assumed in the CQT hypothesis. Uncatalysed rates can then serve as the carbon quality index that represents the inherent chemical recalcitrance of different reactions. In intact soils, however, physical protection provides an additional carbon stabilisation mechanism to determine the overall stability of organic matter in the soil. The actual temperature dependence of decomposition in soils, therefore, will depend on the combined effect of two independent processes, that is, microbial decomposition and adsorption/desorption, which correspond to chemical recalcitrance and physical protection, respectively. The interaction between the two concomitant, yet thermodynamically independent processes (Numa et al., 2021; Pignatello, 1999; Ten Hulscher & Cornelissen, 1996), could thus lead to contrasting conclusions from different experiments (Conant et al., 2011). The combined overall effect can then vary with the relative importance and contribution of the two processes.

## 7 CONCLUSIONS

In summary, our analysis of carbon quality and temperature sensitivity does not support the CQT hypothesis for microbial decomposition of unprotected soil carbon. Regardless of chemical quality, the temperature sensitivity of the enzymatic decomposition of unprotected soil carbon remains similar. This finding suggests that the microbial decomposition of chemically recalcitrant soil carbon is unlikely to respond to warming more strongly than that of labile carbon. However, under a warming climate, the decomposition rate of both recalcitrant and labile carbon will be enhanced, leading to the attendant release of more carbon from soils into the atmosphere (García‐Palacios et al., 2021). But, contrary to the assertion of the CQT hypothesis, that risk does not appear to be further amplified by a heightened temperature sensitivity of the chemically more recalcitrant fractions of soil carbon.

## AUTHOR CONTRIBUTIONS

Lìyǐn L. Liáng, Miko U. F. Kirschbaum, Vickery L. Arcus, and Louis A. Schipper conceived and developed the ideas through countless discussions. Lìyǐn L. Liáng corrected and analysed the data with support from Miko U. F. Kirschbaum, Vickery L. Arcus, and Louis A. Schipper. Lìyǐn L. Liáng wrote the manuscript with contributions from all authors.

## CONFLICT OF INTEREST

The authors declare no competing financial interests.

## Supporting information


Appendix S1.

## Data Availability

The data that support the findings of this study are openly available in Github at https://doi.org/10.5281/zenodo.7346803.
