# Supplementary material for: The carbon‐quality temperature hypothesis: Fact or artefact?
Source: Glob Chang Biol. 2022 Nov 30;29(4):935–42. doi: 10.1111/gcb.16539 (PMC10099867; doi:10.1111/gcb.16539)
Supplement: Supplementary file 1 — Appendix S1. [file GCB-29-935-s001.pdf]

Supplementary Materials for

The carbon-quality temperature hypothesis: Fact or artefact

Lìyǐn L. Liáng[1*], Miko U.F. Kirschbaum[1], Vickery L. Arcus[2], Louis A. Schipper[2]

Correspondence to: LiangL@LandcareResearch.co.nz

This PDF file includes:

Figs. S1 to S3

Table S1

Supplementary Text

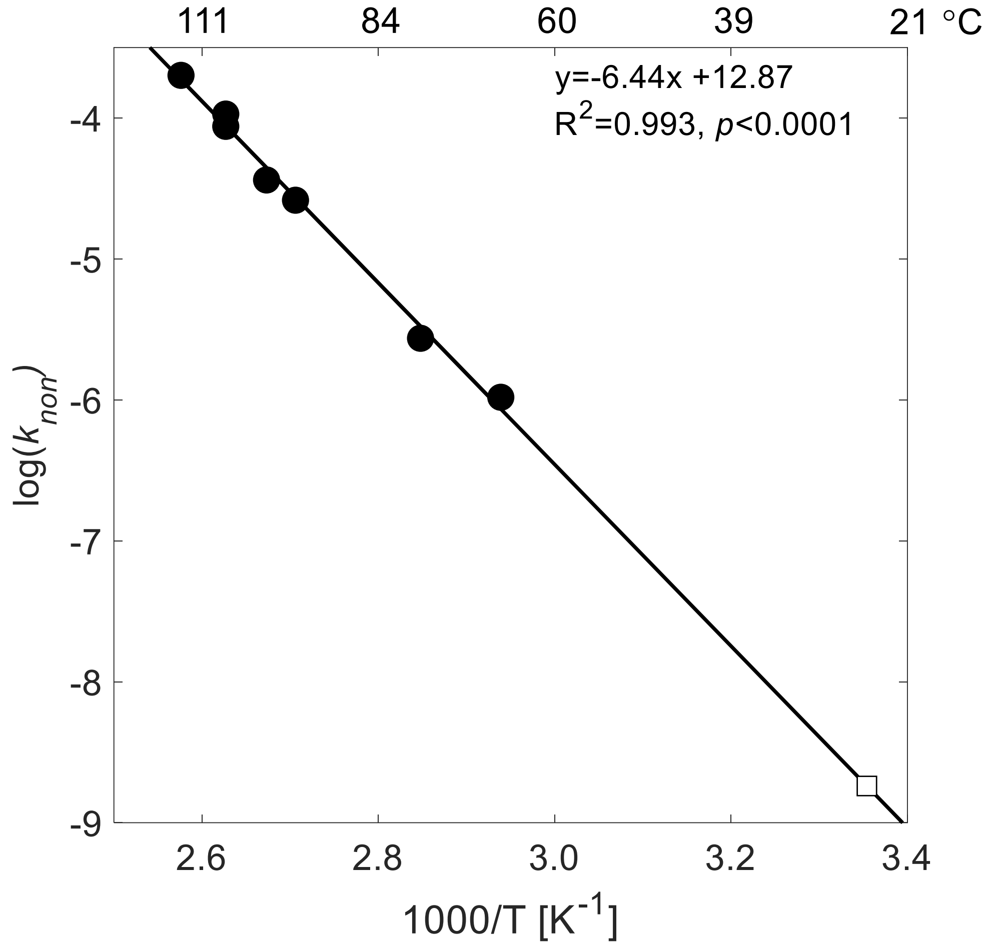

Fig. S1 An example to determine k_{non} at an ambient temperature of 25°C using the Arrhenius plot of glucose decomposition in the absence of enzymes. Rate constants were measured under elevated temperatures (closed circles). The extrapolated value at 25°C (the open square) was used to define the carbon quality of glucose. The regression slope of -6.44 corresponds to an activation energy (E_a) of 123.3 kJ/mol or enthalpy of activation ($\Delta H^{\ddagger}$) of 122.7 kJ/mol ($\Delta H^{\ddagger} = E_a - RT$). The logarithm base 10, instead of the natural base, was used to match the originally reported data. Data were collected from Wolfenden (Wolfenden & Yuan, 2008) by digitising the published figure using WebPlotDigitizer(Rohatgi, 2020).

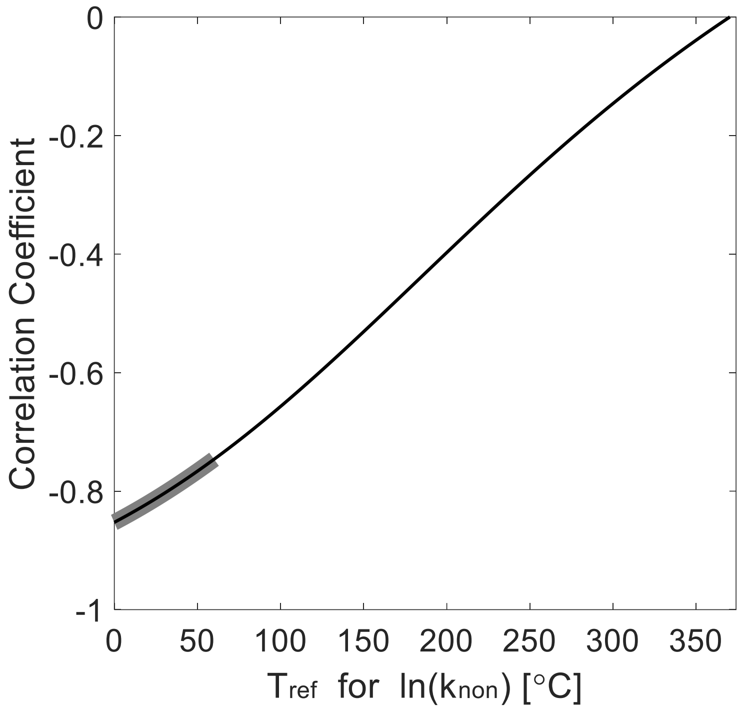

Fig. S2 Correlations between *Q_{10}* and *$ln(k_{non})$* at different temperatures. Correlation coefficient between Q_{10} and $ln(k_{non})$ as a function of reference temperatures between 0 and 374 °C (between the freezing point and the critical temperature of water) for 56 studied uncatalysed reactions, including hydrolysis of disaccharide, polysaccharide, peptides, carboxylic acid amides, etc., decarboxylation of amino acids, orotidine 5'-phosphate, etc. and hydrolytic deamination of cytidine, adenosine, deoxyadenosine, etc. The grey band indicates the biological temperature range of 0 to 60 °C. Like the pattern shown for soil respiration in Fig. 1e in the main text, the correlation coefficient between Q_{10} and $ln(k_{non})$ can shift with the chosen reference temperature between 0 and 374 °C from significant negative correlations at low temperatures, to no correlations at the cross temperatures. Positive correlations are expected at high temperatures above 374 °C. For uncatalysed reactions, the cross temperatures are outside the biological temperature range of 0 to 60°C (grey band), leading to a consistent negative correlation between Q_{10} and $ln(k_{non})$ within the biological temperature range.

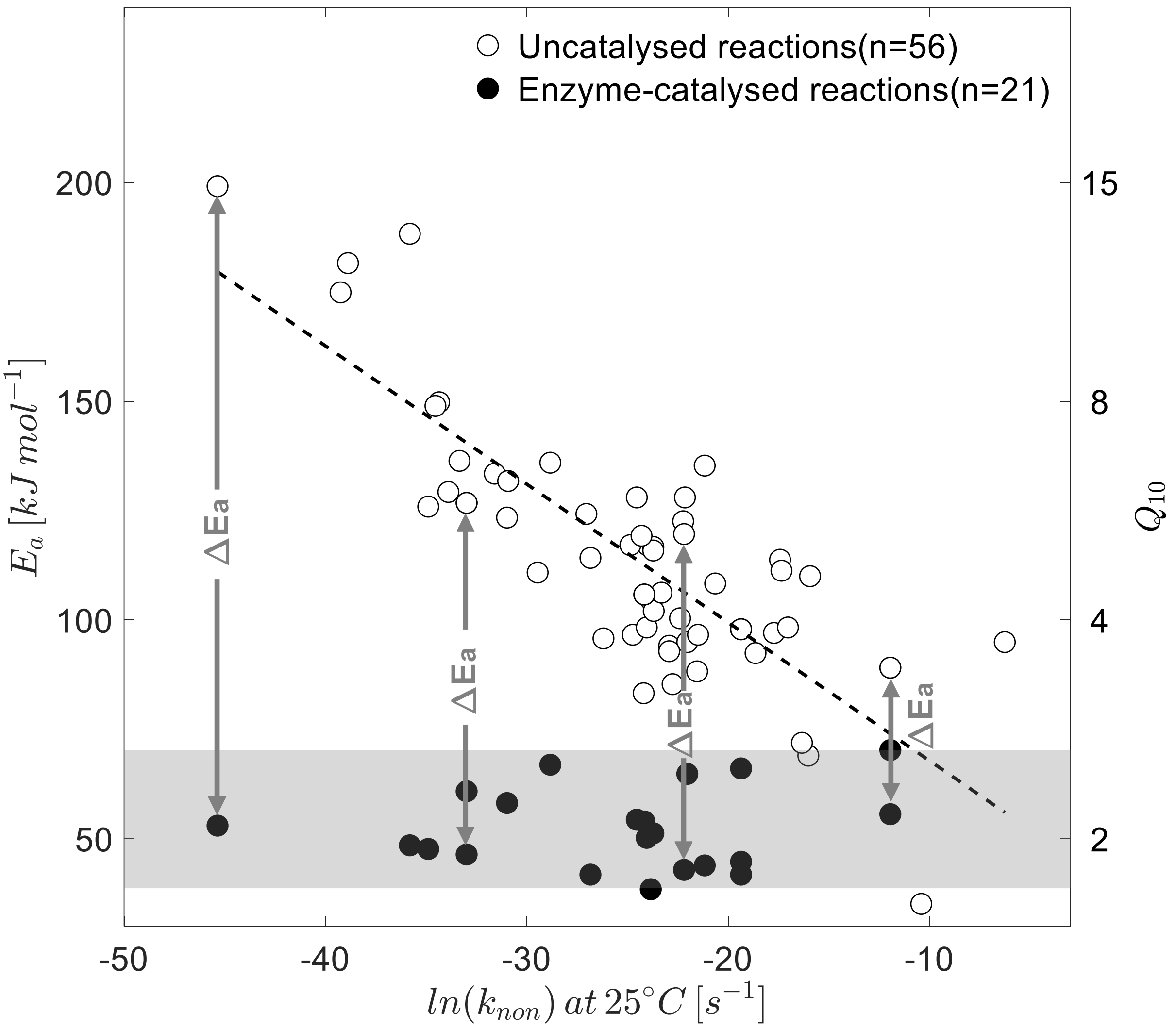

Fig. S3 The correlation between *ln(k*$_{non}$*)* at 25 °C and *E*$_a$ of uncatalysed (open circles) and the corresponding enzyme-catalysed (closed circles) reactions. The right y-axis refers to the corresponding Q_{10} between 20 and 30 °C. The vertical lines indicate reductions of E_a (ΔE_a) from selected uncatalysed to the corresponding enzyme-catalysed reactions, including hydrolysis of fructose-1,6-bisphosphate dianions, β-methyl glucopyranoside and glucose, and rearrangement of chorismate to prephenate, in order from left to right (see values listed in Table S1). The difference in catalytic power among enzymes by reducing E_a could contribute to the loss of tendency between E_a and ln(k_{non}).

Table S1 Comparison of rate constants at 25℃ and activation energies (E_a) of selected uncatalysed and corresponding enzyme-catalysed reactions, with the rate enhancements (k_{cat}/k_{non}) and E_a reduction (ΔE_a) from uncatalysed reactions to enzyme-catalysed reactions.

**Substrate**	**k_{non} [s^{-1}]**	**nE_a [kJ mol^{-1}]**	**Enzyme**	**k_{cat} [s^{-1}]**	**cE_a [kJ mol^{-1}]**	**k_{cat}/k_{non}**	**ΔE_a [kJ mol^{-1}]**
Methyl phosphate dianion	2.0×10^{-20}	199.1	Alkaline phosphatase	60.3	53.0	3.0×10^{21}	146.1
β-1-methylglucopyanoside	4.7×10^{-15}	126.7	Glucosidase	246.4	53.6	5.2×10^{16}	73.1
Glucose	2.3×10^{-10}	119.6	Glucokinase	0.1	42.9	3.3×10^{8}	76.7
Chorismite	6.5×10^{-6}	89.1	Chorismate mutase	3.5	62.9	4.2×10^{6}	26.2

*k_{non}: the rate constant of uncatalysed reactions at 25℃; k_{cat}: the rate constant of enzyme-catalysed reactions at 25℃; nEa: activation energy of uncatalysed reactions; cEa: activation energy of enzyme-catalysed reactions; $\Delta E_a = nE_a - cE_a$.

Supplementary Text

The loss of negative correlation for enzyme-catalysed reactions

Enzymes can accelerate specific reactions to proceed within the time frame of life (~seconds). Enzyme-catalysed rates (k_{cat}) can range about 10^4-fold (Wolfenden, 2011) while uncatalysed reaction rates vary 10^{19}-fold from 10^{-1} to 10^{-20} s^{-1}. Different enzymes therefore have widely different rate enhancements (k_{cat}/k_{non}) in catalysing chemical reactions (Radzicka & Wolfenden, 1995).

For example, k_{cat} of the hydrolysis of fructose-1,6-bisphosphate dianions is 60 s^{-1} while the corresponding k_{non} is 2.0×10^{-20} s^{-1} at 25 °C (Craig, Arriaga, Wong, Lu, & Dovichi, 1996), resulting in a 3.0×10^{21}-fold rate enhancement (k_{cat}/k_{non}) . Correspondingly, the activation energy (E_a) decreases from 199.1 kJ mol^{-1} in the uncatalysed reaction to 53.0 kJ mol^{-1} in the catalysed reaction, with a reduction of 146.1 kJ mol^{-1} in E_a (Table S1). In contrast, for the hydrolysis of β-methyl glucopyranoside and glucose, E_a of the enzyme-catalysed reactions was reduced by only 73.1 and 76.7 kJ mol^{-1}, respectively (Table S1), which were about half of the E_a reduction of alkaline phosphatase. For the rearrangement of chorismate to prephenate catalysed by chorismate mutase, the E_a reduction was even only 26.2 kJ mol^{-1}, about 20% of that reduced by alkaline phosphatase (visualised as vertical lines in Fig. S3).

The magnitude of E_a reduction by different enzymes could vary in proportion to the extent of catalytic enhancement (Table S1, Fig. 2a) to ultimately result in a relatively narrow range of E_a for enzyme-catalysed reactions despite the wide range of E_a values of the uncatalysed reactions. This could have possibly led to the disappearance of the correlation between $ln(k_{non})$ and E_a in enzyme-catalysed reactions. However, enzyme could achieve the rate enhancement not only by reducing E_a, or more specifically the enthalpy of activation (Stockbridge, Lewis, Yuan, &

Wolfenden, 2010; Wolfenden, 2006) ($\Delta H^{\ddagger} = Ea\text{-}RT$) but also increasing the entropy (Jencks, 1975, 1997) ($\Delta S^{\ddagger}$) of activation. If there is no difference in $\Delta H^{\ddagger}$ between uncatalysed and enzyme-catalysed reactions, or both reactions are entropy-driven, the negative correlation between carbon-quality as $ln(k_{non})$ and E_a in uncatalysed reactions could propagate to enzyme-catalysed reactions. It seems to be that reducing $\Delta H^{\ddagger}$ predominates in catalysed reactions with pronounced rate enhancements (Stockbridge et al., 2010; Wolfenden, 2014), rather than increase $\Delta S^{\ddagger}$. Therefore, it could result in the loss of negative correlation between $ln(k_{non})$ and E_a in enzyme-catalysed reactions.

References:

Craig, D. B., Arriaga, E. A., Wong, J. C. Y., Lu, H., & Dovichi, N. J. (1996). Studies on single alkaline phosphatase molecules: Reaction rate and activation energy of a reaction catalyzed by a single molecule and the effect of thermal denaturation - The death of an enzyme. *Journal of the American Chemical Society*, *118*(22), 5245–5253. https://doi.org/10.1021/ja9540839

Jencks, W. P. (1975). Binding Energy, Specificity, and Enzymic Catalysis: The Circe Effect. In *Advances in Enzymology - and Related Areas of Molecular Biology* (pp. 219–410). John Wiley & Sons, Ltd. https://doi.org/10.1002/9780470122884.ch4

Jencks, W. P. (1997). From chemistry to biochemistry to catalysis to movement. *Annual Review of Biochemistry*, *66*, 1–18. https://doi.org/10.1146/annurev.biochem.66.1.1

Moinet, G. Y. K., Hunt, J. E., Kirschbaum, M. U. F., Morcom, C. P., Midwood, A. J., & Millard, P. (2018). The temperature sensitivity of soil organic matter decomposition is constrained by microbial access to substrates. *Soil Biology and Biochemistry*, *116*, 333–339. https://doi.org/10.1016/J.SOILBIO.2017.10.031

Radzicka, A., & Wolfenden, R. (1995). A proficient enzyme. *Science*, *267*(5194), 90–93. https://doi.org/10.1126/science.7809611

Rohatgi, A. (2020). WebPlotDigitizer. Retrieved from https://automeris.io/WebPlotDigitizer/

Stockbridge, R. B., Lewis, C. A., Yuan, Y., & Wolfenden, R. (2010). Impact of temperature on the time required for the establishment of primordial biochemistry, and for the evolution of enzymes. *Proceedings of the National Academy of Sciences of the United States of America*, *107*(51), 22102–22105. https://doi.org/10.1073/pnas.1013647107

Wolfenden, R. (2006). Degrees of difficulty of water-consuming reactions in the absence of

enzymes. *Chemical Reviews*, *106*(8), 3379–3396. https://doi.org/10.1021/cr050311y

Wolfenden, R. (2011). Benchmark reaction rates, the stability of biological molecules in water, and the evolution of catalytic power in enzymes. *Annual Review of Biochemistry*, *80*, 645–667. https://doi.org/10.1146/annurev-biochem-060409-093051

Wolfenden, R. (2014). Massive thermal acceleration of the emergence of primordial chemistry, the incidence of spontaneous mutation, and the evolution of enzymes. *Journal of Biological Chemistry*, *289*(44), 30198–30204. https://doi.org/10.1074/jbc.R114.567081

Wolfenden, R., & Yuan, Y. (2008). Rates of spontaneous cleavage of glucose, fructose, sucrose, and trehalose in water, and the catalytic proficiencies of invertase and trehalas. *Journal of the American Chemical Society*, *130*(24), 7548–7549. https://doi.org/10.1021/ja802206s
